# Seamless Indoor and Outdoor Navigation Using IMU-GNSS Sensor Data Fusion

**DOI:** 10.3390/s26072215

**Published:** 2026-04-03

**Authors:** Bismark Kweku Asiedu Asante, Hiroki Imamura

**Affiliations:** Department of Information Systems Engineering, Faculty of Science and Engineering, Soka University, Tokyo 192-8577, Japan

**Keywords:** multi-sensor fusion, extended Kalman filter, physics informed neural networks, indoor-outdoor navigation

## Abstract

Seamless localization across indoor and outdoor environments remains a fundamental challenge for wearable navigation systems, particularly those intended to assist visually impaired individuals. This challenge arises from the unreliability of GNSS signals in indoor and transitional spaces and the cumulative drift inherent to IMU–based dead reckoning. To address these limitations, this paper proposes a physics-informed GNSS–IMU sensor fusion framework that enables robust, real-time wearable navigation across heterogeneous environments. The proposed system dynamically adapts to environmental context, employing GNSS dominant localization in outdoor settings and PINN enhanced IMU-based dead reckoning during GNSS denied indoor operation. At the core of the framework is a tightly coupled Physics-Informed Neural Network (PINN) and Extended Kalman Filter (EKF), where the PINN embeds kinematic motion constraints to correct inertial drift and suppress sensor noise, while the EKF performs probabilistic state estimation and sensor fusion. The framework is implemented on a compact, energy-efficient wearable platform and evaluated using real-world indoor–outdoor pedestrian trajectories. Experimental results demonstrate improved localization accuracy, significantly reduced drift during indoor navigation, and stable indoor–outdoor transitions compared to conventional GNSS–IMU fusion methods. The proposed approach offers a practical and reliable solution for wearable assistive navigation and has broader applicability in smart mobility and autonomous wearable systems.

## 1. Introduction

Accurate and continuous localization is fundamental to wearable navigation systems developed to support visually impaired individuals. Such systems must operate reliably across diverse environments, including outdoor spaces (e.g., sidewalks, campuses, and urban streets) and indoor settings (e.g., buildings, transportation hubs, and commercial facilities) [1]. However, seamless navigation across these environments remains a persistent challenge due to the inherent limitations of commonly used positioning sensors.

The Global Navigation Satellite System (GNSS) is widely used for outdoor navigation owing to its global coverage and absolute positioning capability. Despite its effectiveness in open environments, GNSS performance deteriorates significantly in indoor spaces and urban canyons due to signal attenuation, multipath interference, and complete signal blockage [2,3]. Consequently, GNSS only solutions fail to provide continuous localization in scenarios involving frequent indoor–outdoor transitions. In contrast, Inertial Measurement Units (IMUs), which estimate motion using accelerometers and gyroscopes, enable infrastructure-free localization and are particularly suitable for indoor navigation [4,5,6]. Nevertheless, IMU-based dead reckoning suffers from cumulative drift caused by sensor noise, bias, and numerical integration errors, resulting in rapidly increasing position errors over time, and several approaches are proposed to address the issue [7,8].

To address these complementary limitations, multi-sensor fusion techniques that integrate GNSS and IMU measurements have been widely investigated [9,10,11,12]. While such approaches improve localization robustness, many existing systems assume continuous or near-continuous GPS availability and do not explicitly account for prolonged GNSS outages or dynamic environmental transitions. In wearable assistive navigation, localization failures during these transitions can negatively impact safety, usability, and user trust [13].

Integrating neural networks into inertial motion navigation has revolutionized the correction of measurement errors by moving beyond traditional, rigid mathematical models to adaptive, data-driven frameworks [14]. While IMU-GNSS systems typically rely on Kalman filtering to fuse signals, these conventional methods often struggle to model the complex, non-linear noise and long-term drift inherent in Micro-Electro-Mechanical-System (MEMS) sensors, especially when GNSS signals are lost or degraded. To bridge this gap, advanced architectures such as Convolutional Neural Networks (CNNs) [15] and Long Short-Term Memory (LSTM) networks are now deployed to learn the specific error characteristics of individual sensors in real-time, effectively denoising raw IMU data and predicting pseudo-GPS updates during outages. By identifying intricate spatial and temporal patterns in acceleration and angular velocity, these neural models can achieve error improvements of over 30% and maintain decimeter-level localization accuracy, ensuring robust and reliable navigation even in the most challenging GNSS denied environments [16].

This work proposes a novel IMU-GNSS tightly coupled data fusion framework designed to provide reliable, real-time navigation across both indoor and outdoor environments through adaptive context awareness. The system automatically detects environmental transitions and dynamically switches between GNSS-dominant localization in outdoor settings and IMU-based dead reckoning during indoor operation. A hybrid sensor fusion algorithm based on an Extended Kalman Filter (EKF) [17,18] with a Physics Informed Neural Network (PINN) is employed to integrate sensor measurements while compensating for GPS unavailability and mitigating IMU drift when GPS updates become available. The PINN is designed to model and correct inertial sensor errors by learning bias and velocity dynamics constrained by physical motion laws. Rather than replacing classical filtering, the PINN operates in conjunction with an EKF, providing physics-regularized corrections to inertial measurements that are injected into the EKF prediction step. PINN models are being considered for data fusion tasks [19] since this approach tightens integration and preserves the probabilistic robustness of the EKF while significantly improving its performance in GNSS denied environments.

The proposed framework will be integrated into our already existing wearable navigation devices that utilize a Jetson-based microcontroller and stereo camera system for assisting the visually impaired with navigation [20,21]. While effective, the earlier platform was limited by the complexity in navigation between indoor and outdoor environments. The earlier system runs visual odometry, which requires a lot of computational resources on the computationally constrained device, thereby making it not feasible for real time tracking of the trajectories taken by the user. This limitation has made it necessary to consider other sensors that are capable of providing position and motion data for real time navigation purposes. IMU and GNSS sensors provide the ideal solution to overcoming this challenge.

This proposed approach for seamless indoor and outdoor navigation using GNSS and IMU data fusion aimed at improving the visually impaired’s ability to have systems that can provide navigation assistance between the two environments seamlessly. The designed approach not only aimed at providing a solution but also offered the following contribution:

1. A novel sensor fusion approach that comprises an Extended Kalman Filter and Physics Informed Neural Network for integrating IMU and GNSS data for seamless indoor and outdoor navigation.
2. We formulate a physics-constrained motion function for next-state prediction in a trajectory sequence.
3. An environment-aware switching strategy that adapts sensor fusion behavior in real time.

The resulting navigation platform supports assistive guidance for visually impaired users and can be extended to applications in smart mobility and autonomous navigation [22]. By enabling seamless indoor–outdoor localization, the proposed framework contributes toward more robust, dependable, and context-aware wearable navigation systems.

## 2. Materials and Methods

### 2.1. Problem Formulation

Consider two distinct environments, Environment A (indoor) and Environment B (outdoor), between which a user moves continuously. The goal is to achieve accurate and continuous tracking of the user’s motion across both environments using two complementary sensors: an IMU and a GNSS receiver. Due to environmental constraints, sensor reliability differs significantly between the two environments, requiring adaptive correction and fusion strategies. To address this challenge, we propose a tightly coupled sensor fusion framework that integrates a Physics-Informed Neural Network (PINN) for state estimation with a Kalman filtering technique to correct inconsistencies between the readings of the two sensors.

### 2.2. Overview

**Figure 1:**
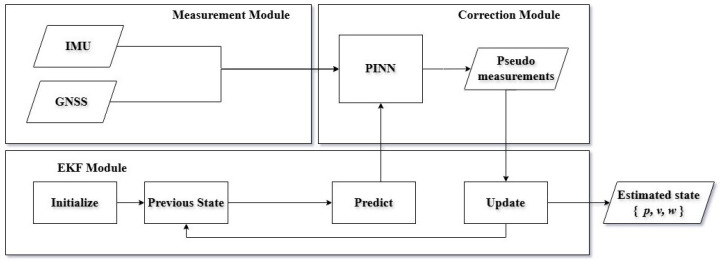
An Illustrated architecture of the proposed hybrid EKF–PINN state estimation framework. Raw IMU and GNSS measurements are processed by a physics-informed neural network (PINN), which incorporates the EKF predicted state to generate physics-consistent pseudo-measurements. These corrections are fused within the EKF update step, improving robustness against sensor noise and modeling errors. The EKF outputs the estimated position, velocity, and orientation.

The proposed approach presents a tightly coupled sensor fusion framework that integrates a Physics-Informed Neural Network (PINN) with an Extended Kalman Filter (EKF) to enable robust and continuous localization using IMU and GNSS measurements. As illustrated in Figure 1, the system consists of three principal modules: (i) multi-sensor data acquisition, (ii) PINN-based correction, and (iii) EKF-based state estimation. The PINN improves sensor reliability by compensating for motion-induced errors, while the EKF performs probabilistic state estimation based on the corrected measurements. The framework mitigates inertial drift, compensates for GNSS inaccuracies, and ensures consistent localization performance across both indoor and outdoor environments.

### 2.3. Multi-Sensor Data Acquisition

**Figure 2:**
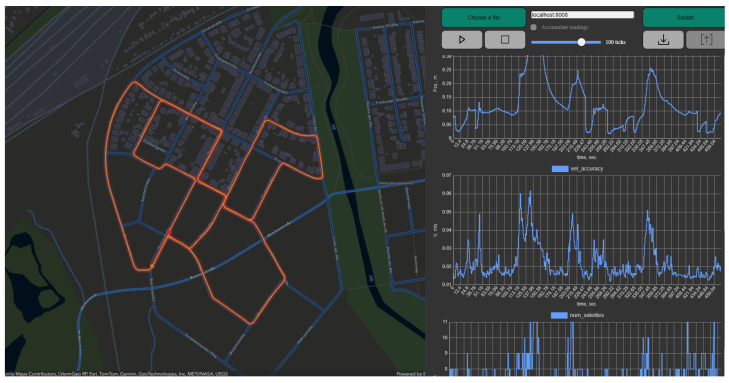
A screenshot of one of the waypoints marked in orange color lines within the KITTI dataset as the ground truth paths in the KITTI IMU-GNSS dataset for training the PINN model displayed using the telemetry app by Andrei Gasparian source: https://github.com/gasparian/telemetry-monitor (accessed on 10 February 2026).

Acquiring the data for this research, we use a publicly available dataset, KITTI IMU-GNSS dataset, which is provided by the Karlsruhe Institute of Technology and Toyota Technological Institute [23] and custom data acquired for our wearable assistive device was developed in earlier research [20]. The publicly available dataset is multimodal sensor data—including stereo camera images, LiDAR point clouds, and IMU/GNSS navigation data recorded from a moving vehicle in Karlsruhe, Germany. Only the IMU/GNSS data component was extracted for this research. The public dataset was used for training the Physics Informed Neural Network. For training the PINN-based correction module, we use a KITTI IMU-GNSS dataset, which is extracted from the raw KITTI dataset [23] for training the model. Figure 2 shows the record paths tracked record by IMU sensors. The available sensor data include inertial measurements (accelerometer and gyroscope), temporal information, and additional active localization signals provided by the indoor positioning infrastructure. These measurements enable continuous tracking of user motion without reliance on passive sensing modalities such as cameras or radar, thereby preserving user privacy and ensuring scalability. These heterogeneous sensor streams serve as inputs to both the correction and filtering stages of the framework. The IMU provides high-rate measurements of linear acceleration and angular velocity, denoted as

(1) $$u_{k}={\left[a_{k},\omega_{k}\right]}^{T}$$

where $a_{k}$ and $\omega_{k}$ represent the acceleration and angular velocity at time step *k*, respectively. The GNSS provides latitude and longitude values, which are converted to absolute position measurements, denoted as

(2) $$z_{k}^{GNSS}={\left[x_{k},y_{k},z_{k}\right]}^{T}$$

where the $x_{k},y_{k}$, and $z_{k}$ represent the latitude, longitude, and altitude at a given time step *k* when satellite signals are available. These measurements are time-synchronized and forwarded to the correction and filtering modules.

The custom data was captured around the campus to test the seamless indoor-outdoor application. The wearable platform, which was developed, was equipped with an IMU and a GNSS receiver. The multi-sensor module continuously collects motion and positioning data from the IMU sensor and the GNSS receiver. The IMU information was extracted from the ZED camera, which is also equipped with the IMU sensor to provide high-frequency acceleration and angular velocity measurements at 400 Hz, enabling fine-grained motion tracking, while the GNSS supplies absolute position updates when available. They were synchronized and aligned to ensure consistent complementarity of the sensory data from the IMU and the GNSS sensors. The GNSS receiver module is equipped with a 25×25×4mm ceramic antenna and supports data acquisition through 50 tracking channels. It processes $L_{1}$ frequency C/A code signals at 1575.42MHz with a high tracking sensitivity of −160dBm. The module enables rapid signal acquisition, achieving a hot start time of 1s and cold or warm start times of 32s. It supports Differential GPS (DGPS), including WAAS, EGNOS, and MSAS, providing a positioning accuracy of up to 3.5m. Position data are computed in the WGS–84 coordinate system and transmitted to Windows-based systems (up to Windows 8) via USB. Additionally, the receiver features a fast recapture time of 0.1s, enabling reliable tracking of dynamic objects with high acceleration (<4 g).

### 2.4. Physics-Informed Neural Network (PINN) for Error Correction

Physics-informed neural networks (PINNs), also known as theory-trained neural networks [24,25], are a class of universal function approximators that explicitly incorporate prior physical knowledge into the learning process. Instead of relying solely on data, PINNs embed governing physical principles typically expressed in the form of differential equations directly into the neural network’s training objective. This integration is particularly advantageous in biological and engineering applications, where acquiring large, high-quality datasets is often impractical [26].

The Physics-Informed Neural Network (PINN) employed in this work is designed to model and correct motion-related sensor errors by embedding physical constraints directly into the learning process. This technique has been tried on some data fusion tasks and showed a promising results [19,27]. The PINN is implemented as a fully connected feedforward neural network that serves as a continuous function approximator for inertial dynamics.

The PINN takes synchronized IMU and GPS measurements as inputs and predicts corrected motion parameters, such as bias-compensated accelerations or velocity updates. By explicitly modeling the underlying physics of motion, the PINN effectively reduces accumulated IMU drift during GPS-denied periods and suppresses noise in GPS measurements during outdoor operation. The predicted corrected next sequence of inputs is then forwarded to the EKF for state propagation and update.

The core of the model is a fully connected multilayer perceptron (MLP) parameterized by θ. The network learns a continuous mapping:

(3) $$f_{\theta }\left(z_{t-1},u_{t-1}\right)=\left\{p\left(t\right),v\left(t\right),\omega \left(t\right)\right\}$$

**Figure 3:**
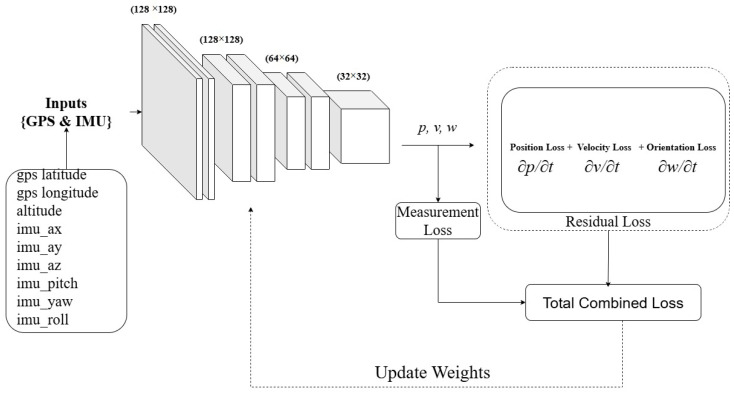
An Illustration of the proposed PINN model for the error correction.

For a given input $z_{t-1}$ and $u_{t-1}$ represent state and control measurements of sensors previous time step t−1. The predicted terms, $p\left(t\right)\in R^{3}$, $v\left(t\right)\in R^{3}$, $\omega \left(t\right)\in R^{3}$ are position, velocity, and angular velocity, respectively, of the next time step *t*, which are approximated using the PINN model as an approximator. The PINN employs a fully connected multilayer perceptron (MLP) composed of four hidden layers with widths 128, 128, 64, and 32 neurons, respectively. The network takes state and control measurement $\left(z_{t-1}\right)$, and control measurement $u_{t-1}$ as input and outputs the trajectory’s next state vector s(t), consisting of position and orientation components, nine (9) outputs in total. All hidden layers use smooth nonlinear activations (tanh), which are well suited for physics-informed learning due to their favorable differentiability properties. Under this configuration, the total number of trainable parameters is 27,335, computed as the sum of affine transformations across layers, including biases. The computational complexity of a single forward pass scales linearly with the number of parameters and is approximately $5.5\times 10^{4}$ floating-point operations (FLOPs), making the model lightweight and suitable for real-time or near–real-time inference. As illustrated in Figure 3, the MLP predicts the position, velocity, and orientation.

#### 2.4.1. PINN Training Strategy

Training Physics-informed neural networks (PINNs) for predicting the next states have proven more challenging than conventional neural networks, with several key issues identified in recent research [28,29]. This research demonstrated that PINNs suffer from spectral bias, struggling to learn complex, high-frequency functions. The IMU and GNSS data are extracted from the KITTI Raw Data [23] partitioned into three non-overlapping subsets for training, constituting 75%, validation 15%, and testing 15% to ensure reliable learning and unbiased evaluation. The split is performed chronologically to preserve the temporal structure of motion trajectories. Network parameters are optimized over 200 training epochs, using the training sets for parameter updates and the validation set to monitor convergence and prevent overfitting. Final performance metrics are reported exclusively on the independent test set.

Model optimization is carried out using the Adam optimizer [30], selected for its stable convergence behavior when training physics-informed neural networks with multiple loss terms. The training objective minimizes a composite loss function defined as

(4) $$L=L_{data}+L_{phys}$$

where the PDE residual loss enforces adherence to the governing physical equations through a mean squared error (MSE) formulation,

(5) $$L_{data}=\frac{1}{N_{r}}\sum_{i=1}^{N_{r}}||N\left(u_{\theta }\left(t_{i}\right)\right){\left|\right|}^{2}$$

Here, N(·) denotes the differential operator of the motion dynamics and $u_{\theta }$ The physics-based motion constraint loss incorporates multiple physically meaningful components to ensure realistic trajectory estimation and is expressed as

(6) $$L_{phy}=\lambda_{p}L_{pos}+\lambda_{o}L_{ori}+\lambda_{a}L_{acc}$$

where the position loss, $L_{pos}$, penalizes discrepancies between predicted and reference positions, the orientation loss, $L_{ori}$, constrains the predicted attitude states, and the acceleration loss, $L_{acc}$, enforces consistency between the second-order derivatives of the predicted position and inertial measurements. The position loss $L_{pos}$ penalizes discrepancies between the predicted position and the reference position obtained from available supervision.

(7) $$L_{pos}=\frac{1}{N_{p}}\sum_{i=1}^{N_{p}}\left|\right|p_{\theta }\left(t_{i}\right)-p^{ref}\left(t_{i}\right){\left|\right|}_{2}^{2}$$

where the $p_{\theta }\left(t_{i}\right)$ and $p^{ref}\left(t_{i}\right)$ term represent the predicted position and actual position of the next points in the trajectory. The orientation loss enforces consistency between the predicted attitude and the reference orientation, represented using unit quaternions.

(8) $$L_{ori}=\frac{1}{N_{o}}\sum_{i=1}^{N_{o}}\left|\right|\omega_{\theta }\left(t_{i}\right)-\omega^{ref}\left(t_{i}\right){\left|\right|}_{2}^{2}$$

where the $\omega_{\theta }\left(t_{i}\right)$ and $\omega^{ref}\left(t_{i}\right)$ term represent the predicted angular orientation of the predicted and the actual data of the next points in the trajectory.

(9) $$L_{acc}=\frac{1}{N_{a}}\sum_{i=1}^{N_{a}}\left|\right|a_{\theta }\left(t_{i}\right)-a^{ref}\left(t_{i}\right){\left|\right|}_{2}^{2}$$

where the $a_{\theta }\left(t_{i}\right)$ and $a^{ref}\left(t_{i}\right)$ term represent the acceleration of the predicted and the actual acceleration data of the next points in the trajectory.

All loss components are formulated using the MSE loss function. The weighting coefficients of the physics-based motion constraint loss were empirically determined to balance the contributions of position, orientation, and acceleration terms. Specifically, the hyperparameters were set to $\lambda_{p}=1.0,\lambda_{o}=0.5,\lambda_{a}=0.1$. These hyperparameter configuration settings reflect the relative reliability and physical significance of each state variable. Position consistency is prioritized due to its direct impact on trajectory accuracy, while orientation and acceleration losses are weighted lower to account for higher sensor noise and modeling uncertainty in inertial measurements.

Model training is conducted on an RTX4080 GPU with the dataset divided into a training dataset and a validation dataset. The training dataset’s performance was used to guide hyperparameter selection and convergence assessment. After training, the final model is evaluated on the independent test set to assess generalization performance, particularly under GPS-available and GPS-denied conditions.

#### 2.4.2. Soft Context Switching via Loss Gating

Soft context switching via loss gating is adopted to handle indoor–outdoor transitions without introducing explicit mode switching or architectural reconfiguration. Instead of discretely classifying the environment as indoor or outdoor, the proposed framework continuously adapts the contribution of measurement-based and physics-based losses during training and inference. When GNSS measurements are available and reliable, the position supervision loss is activated with a higher weight, anchoring the predicted trajectory to absolute global coordinates. During GNSS degradation or complete signal loss, such as in indoor environments or urban canyons, the GNSS-dependent loss terms are smoothly attenuated, while physics-informed motion constraints derived from inertial measurements remain fully active. This loss-gating strategy preserves continuity in the optimization landscape and avoids abrupt state resets or filter reinitialization commonly required in classical switching systems. By embedding context awareness directly into the loss formulation, the PINN learns a unified representation that remains valid across heterogeneous sensing conditions. Importantly, the neural network architecture and state parameterization remain unchanged, ensuring stable gradient flow and consistent temporal dynamics. The approach enables seamless transitions between GNSS-aided and GNSS-denied navigation, reduces sensitivity to misclassification of environmental context, and mitigates error accumulation during extended outages. As a result, the model exhibits robust trajectory estimation performance across indoor, outdoor, and transitional environments while maintaining physical consistency and smoothness in the predicted motion.

To enable seamless indoor–outdoor operation without explicit mode switching, a soft loss-gating mechanism is introduced to modulate the contribution of GNSS-dependent supervision based on measurement availability and quality. The total training loss is defined as

(10) $$L\left(t\right)=\alpha \left(t\right)L_{GNSS}+\left(1-\alpha \left(t\right)\right)L_{phy}+L_{PDE}$$

where $L_{GNSS}$ denotes the position supervision loss derived from GNSS measurements, $L_{phys}$ represents the physics-based motion constraint loss, and $L_{PDE}$ is the governing dynamics residual loss. The scalar gating function α(t)∈[0,1] adaptively controls the relative influence of measurement-driven and physics-driven constraints. The gating function is defined as

(11) $$\alpha \left(t\right)=\sigma \left(\frac{\gamma \left(t\right)-\gamma_{th}}{\tau }\right),$$

where α(·) is the sigmoid function, γ(t) denotes a GNSS quality indicator (we considered the signal-to-noise ratio), $\gamma_{th}$ is a predefined reliability threshold, and τ controls the smoothness of the transition. When GNSS quality is high, α(t)→1, emphasizing absolute position supervision. During GNSS degradation or outage, α(t)→0, causing the optimization to rely predominantly on physics-informed motion constraints. This continuous gating mechanism avoids discontinuities in the loss landscape and enables robust trajectory estimation across indoor, outdoor, and transitional environments.

### 2.5. Extended Kalman Filter Formulation

A given system state x can be estimated using a filtering algorithm such that the position, orientiation, and velocity can be obtained from an IMU, GPS, or a fusion of both. The EKF estimates the system state vector:

(12) $$x_{k}={\left[p_{k},v_{k},\theta_{k}\right]}^{T}$$

where $p_{k},v_{k}$, and $\theta_{k}$ denote position, velocity, and orientation, respectively.

Using the PINN-corrected inertial inputs, the nonlinear state transition model is expressed as:

(13) $$x_{k}^{-}=f\left(x_{k-1},{\overset{-}{u}}_{k}\right)+w_{k},$$

where $w_{k}\;\left(0,Q_{k}\right)$ represents process noise The predicted covariance is computed as:

(14) $$P_{k}^{-}=F_{k}P_{k-1}F_{k}^{T}+Q_{k}$$

where $F_{k}$ is the Jacobian of the state transition function. When GPS measurements are available, the EKF update equations are given by:

(15) $$K_{k}=P_{k}^{-}H_{k}^{T}{\left(H_{k}P_{k}^{-}H_{k}^{T}+R_{k}\right)}^{-1}$$

where $H_{k}$ is the measurement Jacobian and $R_{k}$ denotes measurement noise covariance

The estimated state is fed back as the previous state for the next iteration, enabling continuous real-time operation. During outdoor navigation, GPS updates regularly constrain the filter, while indoors the system relies primarily on PINN-enhanced inertial prediction. This adaptive behavior ensures seamless indoor–outdoor transitions without requiring external infrastructure. Here, we present the approach in the algorithm pseudocode below (Algorithm 1):
**Algorithm 1** Fused IMU-GPS Error Correction Algorithm  1:Input: IMU and GPS measurements, Initial state $x_{0}$, covariance $P_{0}$  2:Output: Estimated state $x_{k}=\left\{p_{k},v_{k},\theta_{k}\right\}$  3:Initialization: Initialize state $x_{0}$ and covariance $P_{0}$  4:Initialization: Initialize EKF noise matrices Q,R  5:**for** each time step k=1,2,3…: **do**  6:     Acquire IMU data $a_{k},\omega_{k}$  7:     Acquire GPS data $z_{k}^{GPS}$ if available  8:     Construct PINN input vector $I_{k}=\left[a_{k},\omega_{k},z_{k}^{GPS}\right]$  9:     Predict the corrected inertial Inputs ${\hat{u}}_{k}=\left[{\hat{a}}_{k},{\hat{\omega }}_{k}\right]$10:   Predict a new state $x_{k}^{-}=\left(x_{k-1},{\hat{u}}_{k}\right)$11:   Predict covariance $P_{k}^{-}=F_{k}P_{k-1}F_{k}^{T}+Q$12:   **if** Is GPS available **then**13:     Compute Kalman gain:     $K_{k}=P_{k}^{-}H_{k}^{T}{\left(H_{k}P_{k}^{-}H_{k}^{T}+R\right)}^{-}$14:     Update State:     $x_{k}=x_{k}^{-}+K_{k}(z_{k}^{GPS}-h\left(x_{k}^{-}\right))$15:     Update covariance:     $P_{k}=\left(I-K_{k}H_{k}\right)P_{k}^{-}$16:   **else**17:     Set $x_{k}=x_{k}^{-},P_{k}=P_{k}^{-}$18:   **end if**19:**end for**20:**return** estimated state $x_{k}$

Combining a physics-informed learning module with probabilistic state estimation, the proposed method for estimating the state of motion combines the strengths of data-driven correction and model-based filtering. The PINN mitigates sensor drift and enforces physical consistency, while the EKF ensures statistically optimal fusion of corrected measurements. This synergy enables accurate, reliable, and wearable-friendly navigation across diverse environments.

### 2.6. Experimental Setup

**Figure 4:**
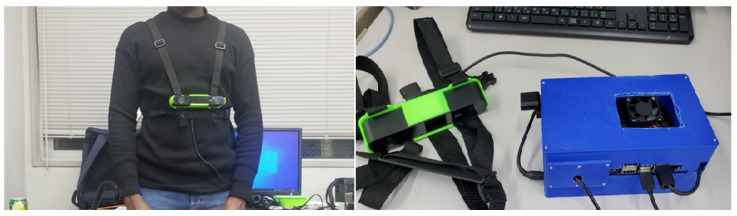
An image of the wearable assistive navigation device which comprise of Zed Camera worn on the chest as in the left image and the device in the right image.

To evaluate the performance of the proposed PINN-assisted GPS–IMU fusion framework, a series of experiments were conducted in mixed indoor–outdoor environments representative of real-world wearable navigation scenarios. The experimental setup consisted of a compact wearable sensing platform equipped with an IMU and a GPS receiver. The collated sensor data were time-synchronized and logged for analysis, while the proposed algorithm was executed in real time using data from our wearable assistive device, which is shown in Figure 4.

The test environments included an outdoor environment, indoor spaces and corridors, and transitional zones such as building entrances, where GPS signal quality degrades faster. The selected areas are selected to assess the robustness and generalization of the framework under intermittent GPS availability and to validate seamless indoor–outdoor operation. Ground truth position data were obtained using reference trajectories derived from high-accuracy positioning sources in outdoor environments and predefined indoor paths with known geometry. All experiments were repeated multiple times to ensure consistency.

#### 2.6.1. Dataset

The evaluation was conducted using a custom wearable navigation dataset collected from a pedestrian walking trajectory of approximately 1.2 km, consisting of alternating indoor corridors and outdoor walkways. The dataset includes synchronized IMU data sampled at 100 Hz and GNSS measurements at 1 Hz. Ground truth was obtained using reference outdoor positioning, which relies on GNSS and predefined indoor routes, which comprise corridors, laboratories, and classrooms of the school building. The dataset reflects an ideal assistive navigation use case and is a representative of daily mobility scenarios.

#### 2.6.2. Evaluation Metrics

To determine the localization, tracking trajectory, and transition smoothness performance of the proposed approach, the PINN-EKF method was quantitatively assessed using the following metrics:

Position Error is the Euclidean distance between the prediction and the ground truth that has been provided and is given by:

(16) $$PE_{k}=∥P_{k}^{est}-P_{k}^{gt}∥$$

where $p_{k}^{est}$ and $p_{k}^{gt}$ denote the estimated and ground-truth positions, respectively.

(17) $$RMSE=\sqrt{\frac{1}{K}\sum PE_{k}^{2}}$$

Also, the drift rate which is the accumulated position error over time during GNSS-denied intervals. The jump error refers to the discrepancy in location data that occurs when switching (transitioning) between different positioning methods used indoors versus outdoors, such as GNSS (outdoors).

The proposed PINN–EKF framework was compared with the following baseline methods: GNSS-only localization, representing conventional outdoor navigation. An IMU only dead reckoning, highlighting inertial drift effects. The approach of inertial navigation considered for the evaluation includes; IMU only does dead reckoning, Standard EKF-based IMU-GNSS fusion, without PINN based correction. The PINN based correction was also investigated and considered in the baseline assessment. These baselines reflect commonly used techniques in wearable and pedestrian navigation systems.

## 3. Results

**Figure 5:**
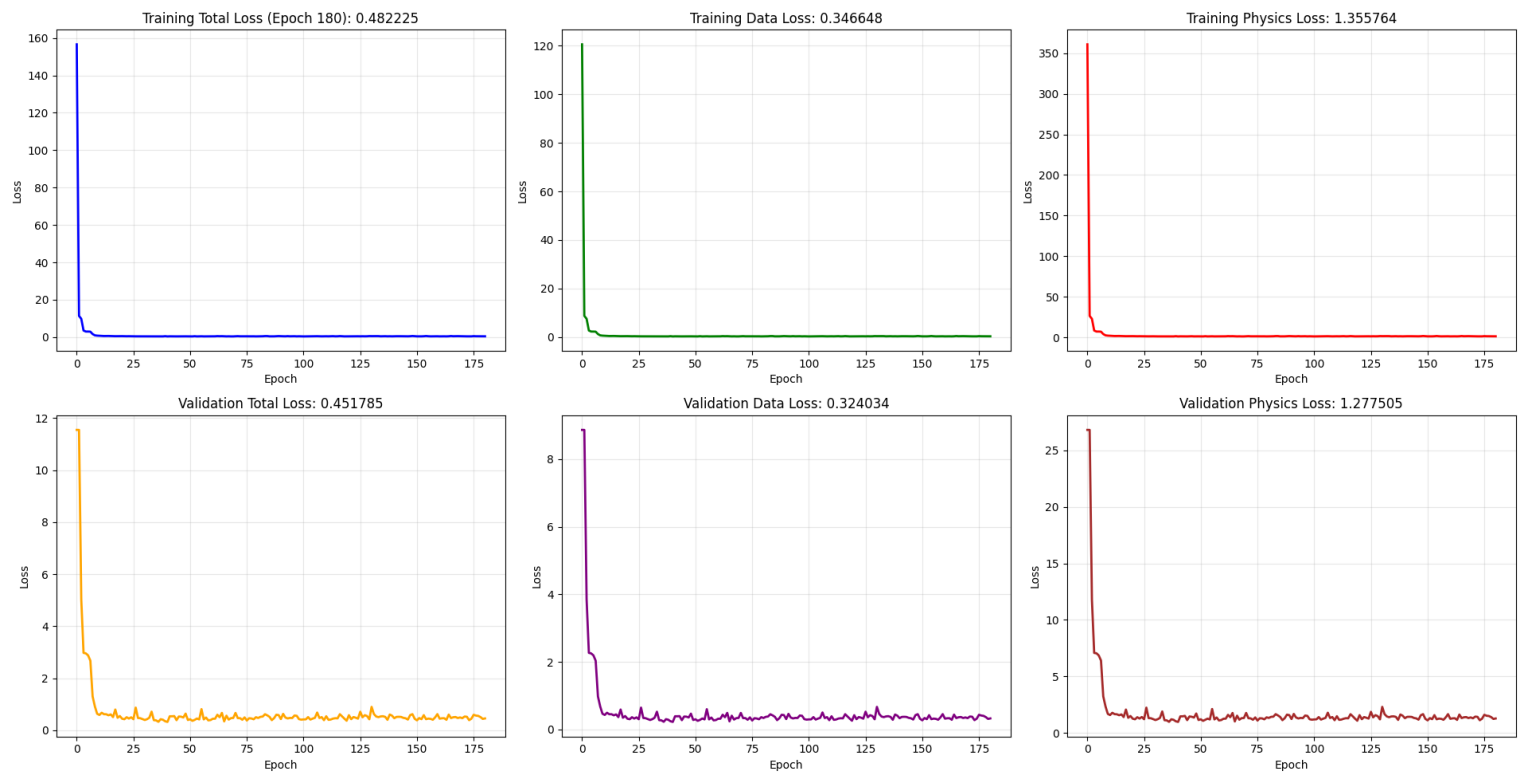
Convergence curve of PINNs’ various losses, the training losses at the top row include the total loss, data loss, and physics loss. The validation loss at the bottom also has the total loss, PDE loss, and physics loss, respectively.

In this section, we present the training results of the proposed Physics-Informed Neural Network (PINN) model, as well as its application to IMU and GNSS sensor data fusion. The PINN is trained to predict the next state in a trajectory sequence. Figure 5 illustrates the convergence behavior of the PINN during training. The plot shows both the training and validation losses, including the data loss and the physics residual loss components. The training and validation curves demonstrate stable and well-converged optimization of the proposed framework. As observed, the total training loss decreases rapidly during the initial epochs, dropping from large initialization values to below 1 within approximately 15–20 epochs, before gradually stabilizing and reaching a final value of 0.482 at epoch 180.

Both the data loss and physics residual loss exhibit similar monotonic decay trends, indicating balanced learning between measurement fitting and physics-based regularization. Importantly, the validation losses closely track the training losses throughout the optimization process. The final validation total loss (0.452) remains consistent with the training performance, with no evidence of divergence or late-epoch growth. The absence of oscillatory behavior and the small generalization gap suggest that the model neither overfits nor underfits the data. Instead, it achieves stable convergence while preserving the imposed physical consistency constraints.

**Figure 6:**
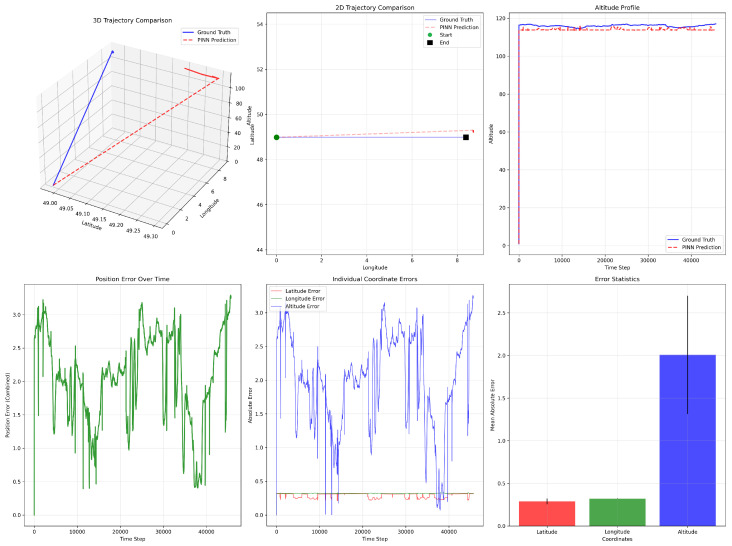
Trajectory comparison between ground truth and PINN predictions. The top row shows the 3D trajectory, 2D horizontal projection, and altitude profile, illustrating close alignment with minor deviations. The bottom row presents the total position error over time, individual coordinate errors, and mean absolute error per axis, highlighting stable horizontal accuracy and comparatively larger altitude estimation errors.

The performance error results shown in Figure 6 demonstrate that the PINN model closely tracks the overall spatial trajectory while exhibiting moderate deviations in specific dimensions. In the 3D and 2D trajectory comparisons (top row), the predicted path follows the general direction and geometric structure of the ground truth, with only minor horizontal displacement errors between the start and end points.

The altitude profile further confirms that the PINN successfully captures the vertical trend and maintains stability over time, although it slightly underestimates the true altitude, resulting in a small bias. This deviation arises because the physics-based loss function was not explicitly designed to compensate for altitude estimation errors, as the primary focus of the model is 2D trajectory reconstruction.

The position error over time (bottom-left) shows that the combined spatial error remains bounded, typically fluctuating between approximately 0.5 m and 3 m without divergence, indicating stable prediction behavior. The individual coordinate error plot reveals that latitude and longitude errors are relatively small and stable (approximately 0.2 to 0.35 m), whereas altitude error dominates the total positioning error, occasionally exceeding 3 m. This is further confirmed by the error statistics bar chart, where the mean absolute error in altitude is significantly larger than that of latitude and longitude. Overall, the model demonstrates strong horizontal positioning accuracy and trajectory consistency, while vertical estimation remains the primary source of residual error.

Table 1 compares the positioning performance of four methods (GNSS-only, EKF, PINN, PINN-EKF) in terms of averaged position error (PE) and root mean square error (RMSE). Method PINN-EKF achieves the lowest error across both metrics, with a PE of 0.4195 m and an RMSE of 0.2692 m, significantly outperforming the other approaches. In contrast, Methods A and C exhibit higher positioning errors (PE = 1.5696 m and 1.2497 m, respectively), indicating larger deviations from ground truth. Method B shows moderate improvement over GNSS-only and PINN (PE = 0.9658 m; RMSE = 0.6564 m), but still underperforms relative to Method PINN-EKF. The consistent reduction in both PE and RMSE suggests that Method PINN-EKF not only decreases average localization bias but also improves overall error stability and robustness. These results demonstrate the effectiveness of the proposed approach in reducing positioning uncertainty compared to conventional baselines.

Table 2 presents a comparative analysis of drift performance across the four evaluated methods in terms of total average drift and drift rate. The GNSS-only and EKF methods exhibit similar degradation behavior, with average drift values of 1.2953 m and 1.2685 m, and corresponding drift rates of 0.1297 m/s and 0.1270 m/s, respectively. These results indicate comparable accumulation of positioning error over time.

The PINN method demonstrates moderate improvement, reducing the average drift to 1.0717 m and the drift rate to 0.1073 m/s, suggesting enhanced stability in motion estimation. In contrast, the proposed PINN–EKF framework significantly outperforms all baseline approaches, achieving a substantially lower average drift of 0.3765 m and a drift rate of 0.0377 m/s. This corresponds to approximately a threefold reduction in drift magnitude compared to the conventional methods.

The consistent improvement observed across both metrics indicates that the PINN–EKF approach effectively mitigates error accumulation and enhances long-term stability, making it particularly suitable for drift-sensitive navigation scenarios, such as GNSS-denied or indoor–outdoor transitional environments.

In Table 3, we present the results of the stability of the indoor-outdoor transition. The maximum jump error, average jump error, recovery time, and the transition were reported.

The results in Table 1, presents the localization performance comparison in the outdoor scenario using averaged position error (PE) and root mean square error (RMSE). Method GNSS-only yields the highest error, with an average PE of 1.5696 m and an RMSE of 0.9872 m. Method PINN shows moderate improvement (PE: 1.2497 m, RMSE: 0.7951 m), while Method EKF further reduces the error to 0.9658 m (PE) and 0.6564 m (RMSE). Method EKF-PINN significantly outperforms all other approaches, achieving the lowest average PE of 0.4195 m and RMSE of 0.2692 m. Compared to the best baseline (Method EKF), Method PINN-EKF reduces RMSE by approximately 59%, demonstrating an improved localization accuracy and robustness in outdoor environments.

**Figure 7:**
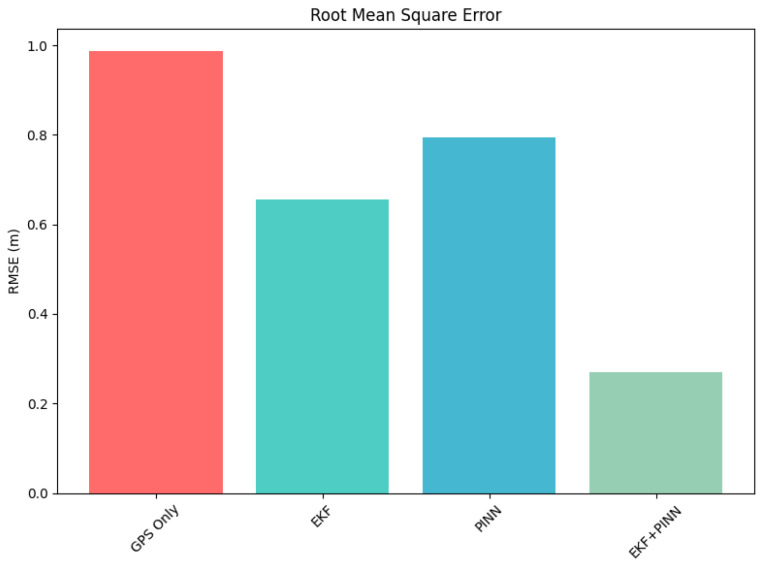
Graph representation of the RMSE of each approach in correcting the measurements obtained from the sensors.

As shown in Figure 7, the combined approach using a Physics-Informed Neural Network and an Extended Kalman Filter yields strong performance in error correction and accurate state estimation when both GNSS and IMU measurements are considered.

**Figure 8:**
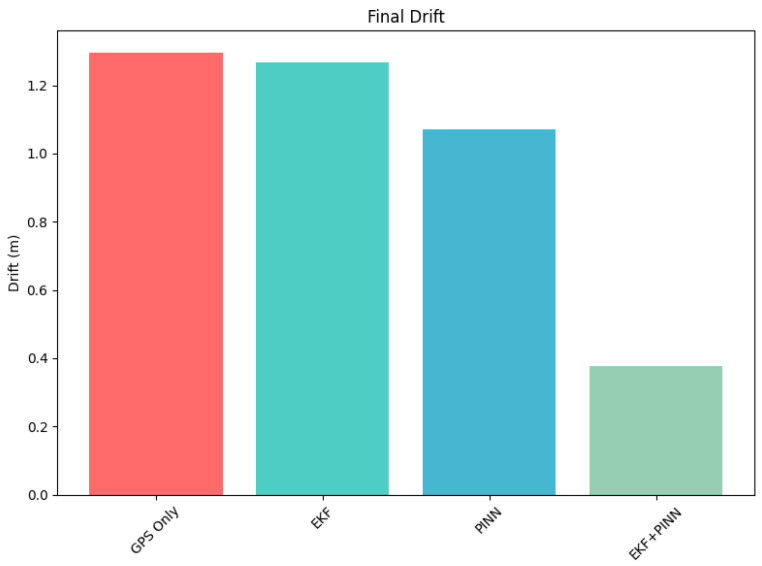
A graph representation of the drift from the positions measurements obtained from the sensors.

**Figure 9:**
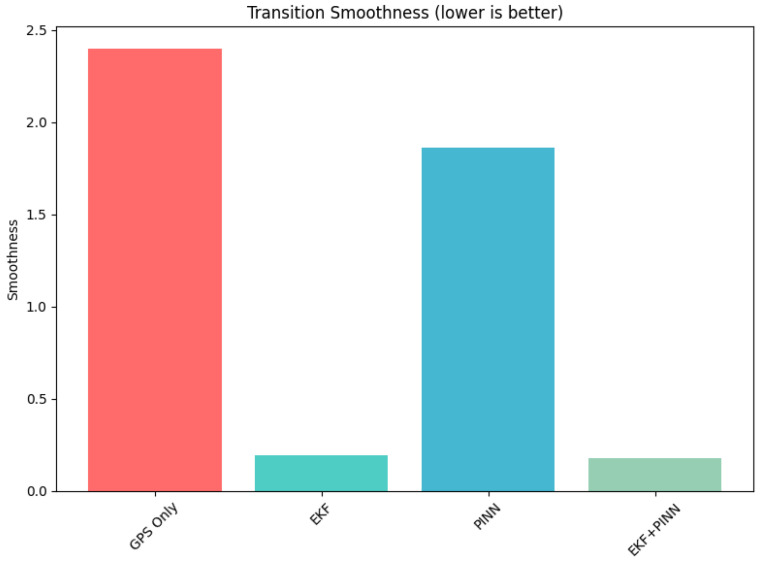
A graph representation of each approach’s transition from indoor to outdoor environment and vice versa.

Figure 8 presents the drift performance of the navigation trajectory using GPS-only, EKF, PINN, and EKF+PINN methods. The incorporation of physics-based constraints results in reduced drift relative to the ground truth trajectory. Figure 9 illustrates the transition between environments. In this case, the EKF+PINN approach maintains a smooth and continuous trajectory as the system moves from one environment to another.

**Figure 10:**
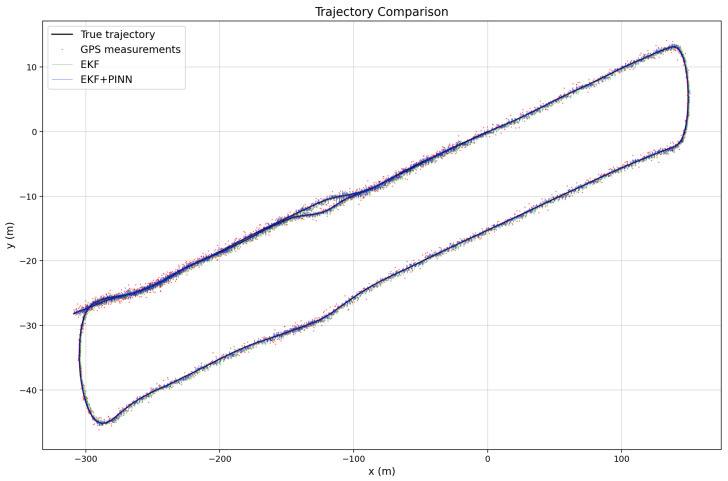
Trajectory comparison for Route 1, showing GPS measurements, EKF estimates, and EKF+PINN results generated from KITTI IMU–GNSS data with simulated noise applied to both IMU and GNSS measurements.

**Figure 11:**
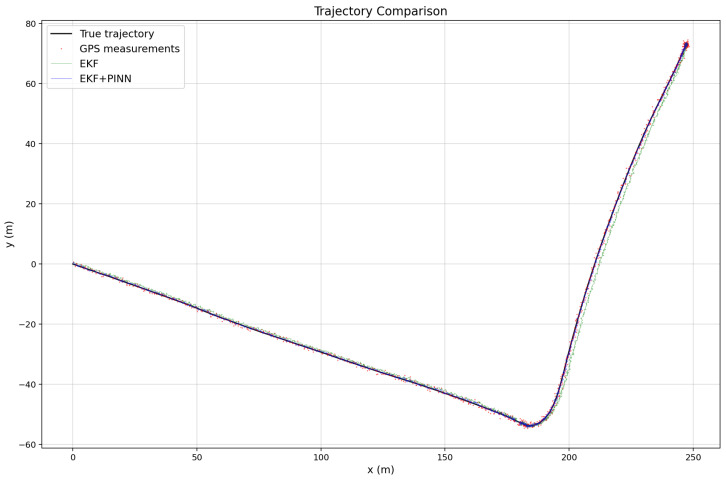
Trajectory comparison for Route 2, illustrating GPS measurements, EKF estimates, and EKF+PINN results under noisy IMU and GNSS conditions.

**Figure 12:**
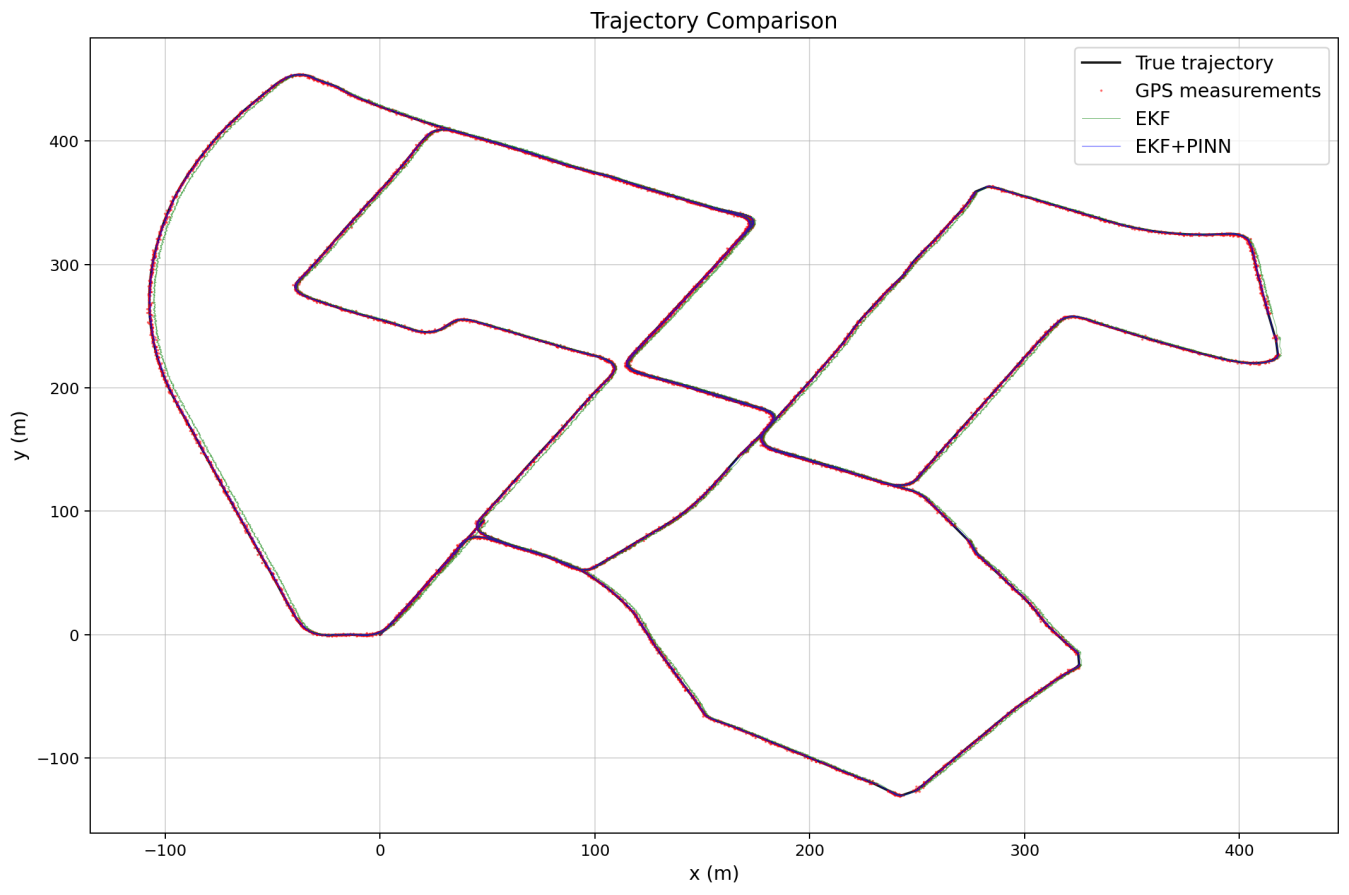
Trajectory comparison for Route 3, demonstrating the improved alignment of EKF+PINN with the reference trajectory compared to GPS-only and EKF estimates.

The three images illustrated in Figure 10, Figure 11 and Figure 12 show the estimated vehicle routes obtained using different sensor fusion frameworks, namely EKF and EKF–PINN, which integrate multi-sensor measurements for robust localization. The GNSS measurements are plotted as red points, while the EKF and EKF–PINN trajectories are shown in green and blue, respectively. The plots demonstrate the effectiveness of the proposed approaches in improving trajectory estimation accuracy.

## 4. Discussion

The experimental results demonstrate that the proposed PINN-assisted EKF framework outperforms conventional EKF fusion methods across outdoor, indoor, and transitional environments. In outdoor scenarios, the proposed method achieves lower RMSE by mitigating GNSS noise and multipath effects through physics-informed correction. Although the improvement over standard EKF-based fusion is moderate under open-sky conditions, the benefit becomes increasingly pronounced in more challenging environments.

In indoor environments and GNSS-denied scenarios, the proposed framework exhibits a reduced drift compared to IMU-only and standard EKF approaches. This improvement is attributed to the PINN’s ability to enforce kinematic consistency and correct inertial bias accumulation during state propagation. As a result, the system maintains bounded error growth over extended periods, which is an important characteristic for wearable navigation applications.

The Indoor–outdoor transition analysis further highlights the robustness of the proposed approach. Unlike baseline methods, which often exhibit abrupt position jumps or delayed convergence upon GNSS re-acquisition, the PINN–EKF framework ensures smooth transitions and rapid stabilization. This property is particularly important for visually impaired users, for whom sudden localization errors can lead to unsafe guidance.

Despite these advantages, the proposed framework has several limitations. First, the current implementation assumes relatively smooth pedestrian motion and does not explicitly model abrupt movements, such as running or sudden turns. Incorporating additional motion models could further enhance robustness under such dynamic conditions. Second, the PINN requires representative training data to generalize effectively across different motion patterns and users. Although training is performed offline, performance may degrade if operational conditions differ significantly from the training scenarios.

Overall, the results confirm that integrating physics-informed learning with probabilistic filtering provides a balanced solution that combines adaptability, physical consistency, and computational efficiency. Despite its advantages, the proposed framework has several limitations. First, the PINN requires representative training data to generalize effectively across different motion patterns and users. While training is performed offline, performance may degrade if the operational conditions differ significantly from the training scenarios.

Finally, by embedding motion dynamics directly into the learning process, the proposed PINN achieves physically consistent correction of inertial measurements, enabling robust integration with EKF-based state estimation under data-limited conditions. Although the computational overhead is suitable for wearable platforms, further optimization may be required for ultra-low-power devices with strict energy constraints.

## 5. Conclusions

This paper presented a PINN assisted IMU-GNSS sensor fusion framework for seamless indoor–outdoor navigation. By combining physics-informed learning with an Extended Kalman Filter for estimating the navigation paths, the proposed approach effectively addresses the limitations of standalone GPS and IMU systems or challenges with the EKF approach. Our experimental results using an indoor location walking dataset and a custom dataset demonstrate improved localization accuracy, reduced inertial drift, and stable indoor–outdoor transitions, all while maintaining real-time performance.

The proposed framework is well suited for integration into assistive navigation systems designed for visually impaired users, where reliability and continuity are important for guiding them to avoid accidents. Beyond assistive technologies, the approach also has potential applications in other navigation systems or applications such as smart mobility, wearable robotics, and context-aware localization systems.

Future works, we will focus on extending the framework to incorporate additional sensing modalities, and vision-based cues for the transition between the indoor and outdoor environments, and on adapting the PINN to support online or semi-online learning. User-specific adaptation and large-scale real-world evaluations will also be explored to further validate the system in diverse operational conditions.

## Figures and Tables

**Table 1:** Localization RMSE Comparison (Outdoor Scenario).

Method	Averaged Position Error PE	RMSE (m)
GNSS-only	1.5696	0.9872
EKF	0.9658	0.6564
PINN	1.2497	0.7951
PINN-EKF	**0.4195**	**0.2692**

**Table 2:** Drift Accumulation during Indoor Navigation (GNSS-Denied, 6 min Walk).

Method	Average Drift (m)	Drift Rate (m/s)
GNSS-only	1.2953	0.1297
EKF	1.2685	0.1270
PINN	1.0717	0.1073
PINN-EKF	**0.3765**	**0.0377**

**Table 3:** Stability analysis during indoor–outdoor transitions.

Method	Max. Jump Error (m)	Ave. Jump Error (m)	Recovery Time (s)	Transition Smoothness
GNSS-only	5.4025	2.2092	0.0700	2.3973
EKF	1.7972	0.0814	0.0060	0.1908
PINN	5.0073	1.6992	0.0114	1.8602
PINN-EKF	**0.8602**	**0.1921**	**0.0030**	**0.1741**

## Data Availability

The data used for this study is a public available dataset which was used for the KITTI Raw Dataset and available at https://www.cvlibs.net/datasets/kitti/raw_data.php (accessed on 20 December 2025).

## Abbreviations

The following abbreviations are used in this manuscript:

CNN	Convolutional Neural Network
DGPS	Differential Global Positioning System
EKF	Extended Kalman Filter
EGNOS	European Geostationary Navigation Overlay Service
GNSS	Global Navigation Satellite System
GPS	Global Positioning System
IMUs	Inertial Measurement Units
LSTM	Long Short Term Memory
MSAS	Multi-functional Satellite Augmentation System
PE	Position Error
PINN	Physics Informed Neural Networks
RMSE	Root Mean Squared Error
SBAS	Satellite Based Augmentation Systems
WAAS	Wide Area Augmentation System

## References

[1] Abidi M.H., Siddiquee A.N., Alkhalefah H., Srivastava V. (2024). A comprehensive review of navigation systems for visually impaired individuals. Heliyon.

[2] Asaad S.M., Maghdid H.S. (2022). A Comprehensive Review of Indoor/Outdoor Localization Solutions in IoT era: Research Challenges and Future Perspectives. Comput. Netw..

[3] Zhuang Y., Sun X., Li Y., Huai J., Hua L., Yang X., Cao X., Zhang P., Cao Y., Qi L. (2023). Multi-sensor integrated navigation/positioning systems using data fusion: From analytics-based to learning-based approaches. Inf. Fusion.

[4] Mallik M., Panja A.K., Chowdhury C. (2023). Paving the way with machine learning for seamless indoor–outdoor positioning: A survey. Inf. Fusion.

[5] Huang F., Zeng A., Liu M., Lai Q., Xu Q. DeepFuse: An IMU-Aware Network for Real-Time 3D Human Pose Estimation from Multi-View Image. Proceedings of the IEEE/CVF Winter Conference on Applications of Computer Vision (WACV).

[6] Hu F., Zheng Q., Ye X., Qiao Z., Xiong J., Chang H. (2025). Gait recognition using spatio-temporal representation fusion learning network with IMU-based skeleton graph and body partition strategy. PLoS ONE.

[7] Brossard M., Barrau A., Bonnabel S. (2020). AI-IMU dead-reckoning. IEEE Trans. Intell. Veh..

[8] Ali R., Liu R., Nayyar A., Qureshi B., Cao Z. (2021). Tightly coupling fusion of UWB ranging and IMU pedestrian dead reckoning for indoor localization. IEEE Access.

[9] Zhu Y., Luo H., Wang Q., Zhao F., Ning B., Ke Q., Zhang C. (2019). A fast indoor/outdoor transition detection algorithm based on machine learning. Sensors.

[10] Liu Q., Gao C., Shang R., Peng Z., Zhang R., Gan L. (2022). Environment perception based seamless indoor and outdoor positioning system of smartphone. IEEE Sens. J..

[11] Cai X., Zhou X., Chen Y., Yang J., Guo F. (2024). A Seamless Indoor-Outdoor Positioning Transition Method Based on HMM/Bayesian Inference Using GNSS and Bluetooth. Proceedings of the 2024 IEEE 100th Vehicular Technology Conference (VTC2024-Fall), Washington, DC, USA, 7–10 October 2024.

[12] Sun X., Zhuang Y., Zheng Z., Zhang H., Wang B., Wang X., Zhou J. (2025). Tightly coupled integration of Visible Light Positioning, GNSS, and INS for indoor/outdoor transition areas. Inf. Fusion.

[13] Barontini F., Catalano M.G., Pallottino L., Leporini B., Bianchi M. (2020). Integrating wearable haptics and obstacle avoidance for the visually impaired in indoor navigation: A user-centered approach. IEEE Trans. Haptics.

[14] Herath S., Yan H., Furukawa Y. (2020). RoNIN: Robust Neural Inertial Navigation in the Wild: Benchmark, Evaluations, and New Methods. Proceedings of the IEEE International Conference on Robotics and Automation (ICRA), Paris, France, 31 May–31 August 2020.

[15] Saeed A.K., Walsh M., Trautman A., Payne D., Gallaher G., Rodriguez B.M. (2025). Ensuring Accurate Navigation Solution in GPS-Denied Scenarios with Machine Learning. Proceedings of the 2025 IEEE Aerospace Conference.

[16] Damagatla R.K.R., Atia M. (2024). Improving EKF based IMU/GNSS fusion using Machine Learning for IMU denoising. IEEE Access.

[17] Ribeiro M.I. (2004). Kalman and extended kalman filters: Concept, derivation and properties. Inst. Syst. Robot..

[18] Caron F., Duflos E., Pomorski D., Vanheeghe P. (2006). GPS/IMU data fusion using multisensor Kalman filtering: Introduction of contextual aspects. Inf. Fusion.

[19] Hu J., Deng C., Zhang Q., Pang A. (2025). Physics-informed neural networks enhanced by data augmentation: A novel framework for robust soil moisture estimation using multi-source data fusion. J. Hydrol..

[20] Asiedu Asante B.K., Imamura H. (2023). Towards robust obstacle avoidance for the visually impaired person using stereo cameras. Technologies.

[21] Asiedu Asante B.K. (2024). Development of a Wearable Assistive Device for Navigation for the Visually Impaired with Command and Request Support. Ph.D. Thesis.

[22] Saadeddin K., Abdel-Hafez M.F., Jarrah M.A. (2014). Estimating vehicle state by GPS/IMU fusion with vehicle dynamics. J. Intell. Robot. Syst..

[23] Geiger A., Lenz P., Stiller C., Urtasun R. (2013). Vision meets Robotics: The KITTI Dataset. Int. J. Robot. Res. (IJRR).

[24] Rad M.T., Viardin A., Schmitz G., Apel M. (2020). Theory-training deep neural networks for an alloy solidification benchmark problem. Comput. Mater. Sci..

[25] Raissi M., Karniadakis G.E. (2018). Hidden physics models: Machine learning of nonlinear partial differential equations. J. Comput. Phys..

[26] Luo K., Zhao J., Wang Y., Li J., Wen J., Liang J., Soekmadji H., Liao S. (2025). Physics-informed neural networks for PDE problems: A comprehensive review. Artif. Intell. Rev..

[27] Rezaei A., Radfar E., Talaeizadeh A., Alasty A. (2024). Physics-Informed Deep Learning-Based Monocular Vision and IMU Fusion for Extracting Position and Heading Data of Multirotors. Proceedings of the 2024 12th RSI International Conference on Robotics and Mechatronics (ICRoM), Tehran, Iran, 17–19 December 2024.

[28] Wang S., Li B., Chen Y., Perdikaris P. (2024). Piratenets: Physics-informed deep learning with residual adaptive networks. J. Mach. Learn. Res..

[29] Wang S., Sankaran S., Wang H., Perdikaris P. (2023). An expert’s guide to training physics-informed neural networks. arXiv.

[30] Kingma D.P., Ba J., Bengio Y., LeCun Y. (2015). 3rd International Conference on Learning Representations.

